# Aseptic Μeningitis in Hereditary Autoinflammatory Diseases

**DOI:** 10.7759/cureus.8244

**Published:** 2020-05-22

**Authors:** Antonis Neokleous, Savvas Psarelis, Konstantinos M Parperis

**Affiliations:** 1 Internal Medicine, University of Cyprus Medical School, Nicosia, CYP; 2 Division of Rheumatology, Nicosia General Hospital, Nicosia, CYP; 3 Division of Rheumatology, University of Arizona College of Medicine, Phoenix, USA

**Keywords:** autoinflammatory diseases, familial mediterranean fever, chronic infantile neurological cutaneous articular syndrome, aseptic meningitis, interleukin 1, interleukin 6, cryopyrin-associated periodic syndrome, neurological manifestations

## Abstract

Autoinflammatory diseases (ADs) refer to a group of disorders of the innate immune system, mainly monogenic, marked by episodes of systemic inflammation. Aseptic meningitis is a rare neurological manifestation of ADs characterized by meningeal inflammation, negative routine cultures in the cerebrospinal fluid and identical signs and symptoms of bacterial meningitis. Herein, the aim of this review article is to describe the association between aseptic meningitis and ADs, especially in patients with familial Mediterranean fever (FMF) and chronic infantile neurological cutaneous articular (CINCA) syndrome. We will discuss the emerging role of proinflammatory cytokines, such as interleukin 1 (IL-1), interleukin 6 (IL-6) and tumor necrosis factor-alpha (TNF-α), in the pathogenesis of aseptic meningitis in ADs, and will explore recent treatment developments, such as the use of biological agents.

## Introduction and background

Autoinflammatory diseases (ADs) are systemic disorders of the innate immune system, characterized by repeated episodes of inflammation, without the presence of autoantibodies or reactive T cells. An overactive innate immune system plays a key role in the pathogenesis of these disorders through the increased production and activity of interleukin 1β (IL-1β) and interleukin 6 (IL-6) [1]. So far, there are 12 known monogenic ADs: (1) familial Mediterranean fever (FMF), (2) tumor necrosis factor receptor-associated periodic (TRAPS) syndrome and the cryopyrin-associated periodic syndromes (CAPS) that comprises the (3) familial cold autoinflammatory syndrome (FCAS), (4) Muckle-Wells syndrome (MWS) and (5) chronic infantile neurological cutaneous articular (CINCA) syndrome. Other ADs include the (6) mevalonate kinase deficiency (MKD), (7) NLRP12-associated autoinflammatory disorder, (8) Blau syndrome (BS), (9) early-onset sarcoidosis, (10) pyogenic arthritis-pyoderma gangrenosum and acne (PAPA) syndrome, (11) Majeed syndrome (MS) and (12) deficiency of the interleukin 1 (IL-1) receptor antagonist. Each of these conditions may manifest itself with a wide array and severity of systemic and organ-specific symptoms and usually they have been associated with elevated inflammatory biomarkers [2]. ADs are generally marked by an early onset in the first year of life or early childhood; however, adult onset has also been described, particularly in FMF and TRAPS [3-5]. Many of these syndromes are hereditary and result from a single gene mutation (Table 1) [5].

**Table 1:** Classification of the Monogenic Autoinflammatory Syndromes FMF: familial Mediterranean fever, MKD: mevalonate kinase deficiency, TRAPS: tumor necrosis factor receptor-associated periodic syndrome, CINCA: chronic infantile neurological cutaneous articular syndrome, FCAS: familial cold autoinflammatory syndrome, MWS: Muckle–Wells syndrome, BS: Blau syndrome, DIRA: deficiency of interleukin 1 receptor antagonist, MS: Majeed syndrome, PAPA:  pyogenic arthritis pyoderma gangrenosum and cystic acne syndrome, NLRP: NACHT domain-, leucine-rich repeat- and pyrin domain-containing protein, AD: autosomal dominant, AR: autosomal recessive [5]

	Gene	Mutated Protein	Inheritance
Monogenic periodic fevers			
FMF	MEFV	Pyrin/marenostrin	AR
MKD	MVK	Mevalonate kinase	AR
TRAPS	TNFRSF1A	TNFRSF1A	AD
Cryoporin-associated periodic syndromes			
CINCA	NRLP3/CIAS1	Cryopyrin	AD, sporadic
FCAS			AD
MWS			AD
NLRP12-associated autoinflammatory disorder	NLRP12	NLRP12	AD
Autoinflammatory granulomatous disorders			
BS	NOD2/CARD15	NOD 2(CARD15)	AD
Early-onset sarcoidosis	NOD2/CARD15	NOD 2(CARD15)	Sporadic
Autoinflammatory pyogenic disorders			
DIRA	IL1RN	Interleukin 1 receptor antagonist	AR
MS	LPIN2	Lipin-2	AR, sporadic
PAPA	PSTPIP1 (CD2BP1)	PSTPIP1 (CD2BP1)	AD

To achieve an accurate diagnosis, a number of steps need to be taken, including detailed history taking, physical exam, laboratory studies and genetic testing. Characteristic clinical features of these disorders include periodic fevers, neutrophilic rashes or urticaria, polyserositis, polyarthralgia or polyarthritis, hepatosplenomegaly, lymphadenopathy, elevated acute phase reactants, neutrophilia and a long-term risk of secondary amyloidosis [6]. Moreover, these diseases may also present with neurological symptoms due to aseptic meningitis [7,8]. The most frequent conditions associated with aseptic meningitis appear to be the CIGNA syndrome, an AD that belongs to the family of the CAPS. In particular, meningitis can be the initial manifestation in FMF and CINCA syndrome; however, it can occur at any stage of the disease. A potential pathophysiologic mechanism highlights the role of IL-1, IL-6 and tumor necrosis factor-alpha (TNF-α) in the pathogenesis of aseptic meningitis in FMF and CINCA syndrome [9-11].

## Review

The pathophysiology of ADs

The immune system contains a complex armamentarium of cells and signaling molecules, which can combat infection, eradicate foreign agents and promote repair. Over time, the immune system, which comprises the innate and adaptive immunity, has evolved to provide effective protection against infectious diseases [12]. The innate immune system is characterized by nonspecific defense mechanisms against foreign antigens soon after their appearance, while the adaptive immunity comprises specific recognition of pathogens before a response is initiated. IL-1, a signaling molecule of the innate immune system and a proinflammatory cytokine, is typically overexpressed following injury or infection, and through its receptor, it may affect a wide variety of cell types within the body [13]. IL-1 belongs to a family of cytokines comprising 11 different isoforms, though most is known about IL-1α and 1β because of their central role in regulation of inflammation. IL-1β is expressed as a precursor protein that is then cleaved by caspase-1, a protein that forms a part of a complex known as the inflammasome, producing the active IL-1β. When the balance of the immune response is distorted, it can promote inflammation and the pathogenesis of various diseases. Overproduction of IL-1β is associated with a variety of autoinflammatory syndromes, specifically FMF and CINCA syndrome. Some are associated with mutations within NACHT domain-, leucine-rich repeat- and pyrin domain-containing protein 3 (NLRP3), which is a gene that encodes cryopyrin, a component of the inflammasome. These mutations are thought to increase the expression of the active interleukin 1β promoting abnormal activity of this signaling molecule that may result in the development of FMF and CINCA syndrome [14].

Aseptic meningitis as a neurological complication in ADs

Previous studies demonstrated that the production of proinflammatory cytokines such as TNF-α, IL-6 and IL-1β by monocytes of the central nervous system (CNS) seems to play an important role in the alteration of the blood-cerebrospinal fluid (CSF) barrier [15,16]. Although the mechanism of action is still not fully defined, experimental studies in meningitis induced by infection suggest that these cytokines are directly cytotoxic to the neurons. Furthermore, the cytotoxicity can occur indirectly, through the release of oxygen free radicals, which attack the double bonds of polyunsaturated fatty acids on cellular membranes in the process of lipid peroxidation of the membrane [17].

The proinflammatory cytokines, together or independently, are being responsible for the first steps of the inflammatory cascade that results in organ damage. Patients with ADs who develop aseptic meningitis present with classic symptoms of meningeal inflammation, including nausea, headache, fever and neck stiffness. The clinical course of aseptic meningitis varies from acute, self-limited episode to chronic or recurrent disease, and when meningitis is the initial manifestation of the AD it might pose a diagnostic challenge [18]. The diagnosis is based on the characteristic signs and symptoms of meningitis, after ruling out alternative diseases. It is critical for health care providers to exclude other etiologies such as a bacterial or fungal infection or the use medications that can induce aseptic meningitis, for example nonsteroid anti-inflammatory drugs [19]. The diagnostic evaluation includes lumbar puncture with CSF analysis, electroencephalogram, evoked-potential studies, CT and MRI of the brain. The results of these studies will allow the clinician to thoroughly evaluate the patient with symptoms of meningitis [20]. For the patients who experienced persistent symptoms of aseptic meningitis associated with fever, and negative infectious diseases workup, we would recommend genetic testing for ADs. In most patients with aseptic meningitis and autoinflammatory syndrome the CSF analysis may yield pleocytosis with elevated protein and normal glucose levels [18].

Aseptic meningitis in FMF

FMF is a monogenic autosomal recessive disease marked by recurrent episodes of fever, polyserositis, arthritis and erysipelas-like erythema [21]. It is caused by mutations in the MEFV gene that encodes the protein pyrin [22,23]. This protein is made up of 781 amino acids and is expressed mostly in neutrophils, eosinophil granulocytes, monocytes, fibroblasts of the skin, peritoneum and synovial. Pyrin mutations cause changes in inflammasome function, which lead to increased synthesis of proinflammatory cytokines, (mainly IL-1β and IL-6) and altered inhibition of apoptosis. MEFV mutation might contribute to the inflammatory phenotype in the presence of other genetic or environmental factors, through IL-6 hypersecretion [24]. Microglia, astroglia or both are the possible sources of IL-6 in the CNS. The CNS hypersecretion of IL-6 may cause increased blood brain-barrier permeability, leading to subtle edema, CSF inflammation with increased intracranial pressure and inner ear damage [25]. 

From the clinical point perspective, different FMF genotype and phenotypes have been identified [26]. The typical FMF phenotype (mutations in MEFV exon 10) is characterized by recurrent inflammatory episodes. During these episodes, patients mainly complain of fever and abdominal pain. Patients with the incomplete FMF phenotype (mutations in MEFV exons 2 or 3) present with uncommon manifestations such as fever and headache due to aseptic meningitis [8,27]. Meningitis, in FMF, follows a similar pattern with the fever, with self-limited episodes, lasting three to five days [23]. In terms of treatment, colchicine is considered as the gold standard for patients with FMF. Other treatment strategies, in patients who fail to respond to colchicine, include the use of biologics, specifically the IL- 1 inhibitors and TNF-a blockers. Examples include the anakinra, an IL-1 receptor inhibitor, the canakinumab, an anti-IL-1β monoclonal antibody, the rilonacept, an IL-1 receptor fusion protein, the infliximab, a TNF-a monoclonal antibody and the etanercept, a TNF dimeric fusion protein [28].

Aseptic meningitis in CAPS and CINCA syndrome

CAPS are a group of ADs transmitted by autosomal dominant inheritance caused by mutations in the NLRP3 gene, encoding cryopyrin, an inflammasome protein that directly activates IL-1β. This mutation induces an overexpression of IL-1β, leading to episodic fever associated with organ-specific inflammatory symptoms. There are three known forms of CAPS: the familial cold auto-inflammatory syndrome (FCAS), the Muckle-Wells syndrome (MWS) and the CINCA syndrome, which is the most severe form (Table 2) [24,29-31].

**Table 2:** Cryopyrin-Associated Periodic Syndrome (CAPS) FCAS: familial cold autoinflammatory syndrome, MWS: Muckle–Wells syndrome, CINCA: chronic infantile neurological cutaneous articular syndrome, AD: autosomal dominant, AA: amyloid A [24]

FCAS	MWS	CINCA
Inheritance		
AD	AD	AD, sporadic
Main clinical features		
Cold-induced:	Fever	Fever
-Fever	Urticarial rash	Urticarial rash
-Rash	Conjuctivitis	Conjuctivitis
-Conjunctivitis	Arthralgia	Visual and intellectual damage
-Arthralgia	Sensorineural deafness	Sensorineural deafness
	AA amyloidosis (in 25% of patients) leading to renal failure	Progressive chronic meningitis
		Destructive arthritis
		AA amyloidosis leading to renal failure

In a cohort study by Parker et al., the investigators showed that three out of four patients with CAPS with available CSF data had evidence of aseptic meningitis [32]. Although meningitis can occur in all three types of CAPS, meningitis in CINCA syndrome patients is more common. CINCA syndrome may manifest with fever, urticaria, sensorineural hearing loss, uveitis, lymphadenopathy, hepatosplenomegaly papilledema, optic nerve atrophy leading to blindness, mental retardation, deforming osteoarthropathy of the large joints, hypertrophy of growth plates and chronic meningitis [32]. Chronic aseptic meningitis, defined as at least one month of unresolved inflammation of the CSF, might result in increased intracranial pressure, ventriculomegaly, cerebral and optic nerve atrophy. Given that elevated levels of IL-1 play an important role in the disease pathogenesis of CAPS, clinician can consider the use IL-1 inhibitors in order to control inflammation. Anakinra was the first drug utilized in these patients, resulting in significant improvement in neurological symptoms [33-35]. Furthermore, a follow-up study showed that in patients treated with anakinra, the CSF levels of cytokines IL-1 and IL-6 and monocyte and granulocyte counts were reduced significantly [31]. Alternatively, in patients who failed or did not tolerated anakinra, canakinumab and rilonacept monoclonal antibodies against IL-1β, might be alternative therapeutic options [11,36-38]. To our knowledge, there are no approved biologic disease-modifying antirheumatic drugs for aseptic meningitis in ADs.

## Conclusions

The pathogenetic mechanisms of aseptic meningitis in ADs remain unclear; thus, no consensus exists regarding the optimal treatment and prognosis. Future studies are required to elucidate pathophysiology of aseptic meningitis associated with ADs in order to develop treatment strategies, and improve patient outcomes and prognosis. Physicians should be aware of this manifestation to identify early the disease and treat appropriately. From a therapeutic point of view, no evidence-based therapy exists for aseptic meningitis in ADs; however colchicine and corticosteroids are often used to treat symptoms in ADs, with varying results. Recent advances for the development of biological agents, such as anti-TNF-a, anti-IL-1 and anti-IL-6, have opened up new therapeutic options for the management of these heterogeneous disorders.
